# New Insights into Valve Hemodynamics

**DOI:** 10.5041/RMMJ.10400

**Published:** 2020-04-29

**Authors:** Gil Marom, Shmuel Einav

**Affiliations:** 1 School of Mechanical Engineering, Tel Aviv University, Tel Aviv Israel; 2 Department of Biomedical Engineering, Tel Aviv University, Tel Aviv, Israel

**Keywords:** Aortic valve, heart valves, hemodynamics, mitral valve, pulmonary valve, tricuspid valves

## Abstract

Heart valve diseases are common disorders with five million annual diagnoses being made in the United States alone. All heart valve disorders alter cardiac hemodynamic performance; therefore, treatments aim to restore normal flow. This paper reviews the state-of-the-art clinical and engineering advancements in heart valve treatments with a focus on hemodynamics. We review engineering studies and clinical literature on the experience with devices for aortic valve treatment, as well as the latest advancements in mitral valve treatments and the pulmonic and tricuspid valves on the right side of the heart. Upcoming innovations will potentially revolutionize treatment of heart valve disorders. These advancements, and more gradual enhancements in the procedural techniques and imaging modalities, could improve the quality of life of patients suffering from valvular disease who currently cannot be treated.

## INTRODUCTION

Heart valve diseases are common disorders with five million annual diagnoses in the United States alone. In general, these diseases can cause any of the four heart valves to malfunction, mostly as a result of stenosis or regurgitation, and they can only be treated by surgical or transcatheter interventions. Obviously, any heart valve disorder alters its hemodynamic performance; therefore, treatments are aimed at restoring blood flow to healthy conditions. This paper reviews the current valvular treatments from an engineering perspective, with a focus on hemodynamics. This review is inspired by Professor Rafael Beyar’s contributions to cardiac treatments based on engineering principles, his aspiration for research-based innovation,^1^,^2^ and his early research on computational simulations. This review will focus first on the aortic valve, which has been extensively studied and has the most substantial clinical experience with treatment devices. We will then review the latest advancement for the other valves of the heart. This review will conclude with a look at prosthetic valves and the hemodynamic standards to which they must comply.

## THE AORTIC VALVE

There are two main acquired aortic valve disorders, both of which affect cardiac hemodynamics. Aortic root aneurysm is usually the cause of aortic insufficiency, where the valve leaflets cannot fully coapt. Aortic stenosis, on the other hand, is most commonly a result of calcific aortic valve disease (CAVD). Among the congenital disorders, bileaflet aortic valve is the most common. From a hemodynamic perspective, insufficiency is due to a regurgitating valve; hence, the preferred treatment is surgical valve-sparing. This treatment provides physiologic hemodynamic conditions that have a much lower thrombogenic risk than prosthetic valves.^3^ However, the only treatment for the much more commonly occurring CAVD is valve replacement with a prosthetic valve.^4^,^5^

**Figure 1:**
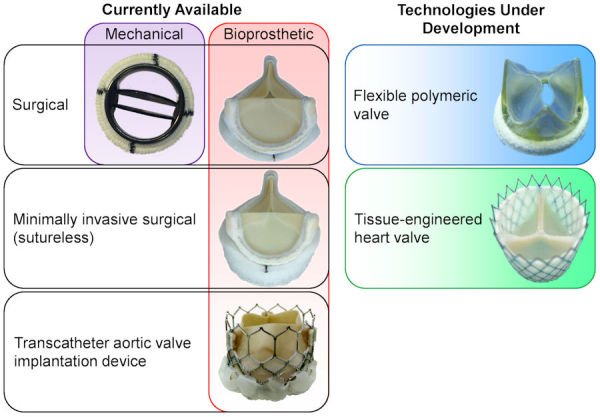
Available (Left) and Under Development (Right) Prosthetic Aortic Valve Technologies Photographs adapted and modified from Figure 5 of Spühler et al.^6^ [CC BY 4.0], Figure 1 of Capelli et al.^7^ [CC BY 4.0], Figure 6 of Ghanbari et al.^8^ [CC BY-NC 3.0], and Figure 1 of Sanders et al.^9^ [CC BY 4.0].

Currently, two types of surgical prosthetic valves are available: mechanical and bioprosthetic (Figure 1, left panel). Mechanical valves are very durable and can be used in artificial hearts^10^; however, their main disadvantage is thrombus formation due to non-physiologic hemodynamics. Bioprosthetic valves, on the other hand, mimic the native valve function, thereby eliminating the long-term anticoagulation requirement,^11^ but they have limited durability compared with mechanical valves. Nevertheless, in recent years the American Heart Association, American College of Cardiology, European Society of Cardiology, and the European Association for Cardio-Thoracic Surgery have updated their guidelines and lowered the recommended patient age for bioprosthetic valves.^12^ One rationale for this change is the extensive use of transcatheter aortic valve implantation (TAVI) and the ability to treat bioprosthetic valve failure with a valve-in-valve (ViV) procedure,^13^,^14^ thereby avoiding repeated open heart surgery. An additional option for rapid deployment of surgical bioprosthetic valves is the use of sutureless aortic valves^15^; however, these valves may increase the risk for complications that are usually associated with TAVI (described in the next section).

The flexible polymeric valve is a developing technology that may potentially combine the strengths of the mechanical and bioprosthetic valves, resulting in physiological hemodynamics with high durability. Moreover, this type of valve can be more cost-effective than a bioprosthetic valve since it will have a lower rejection rate during the manufacturing process.^16^,^17^ There is a long history of attempts to develop polymeric valves, which failed to receive regulatory approval.^18^ Nevertheless, several such devices are currently being developed and have demonstrated promising experimental results.^16^,^19^–^22^ However, even if polymeric valves do achieve the hemodynamic capabilities of current bioprosthetic valves—with the durability of mechanical valves—their inability to grow makes them problematic for pediatric use. Tissue-engineered heart valves may, potentially, be able to adjust to both tissue growth and remodeling, therefore ensuring prolonged durability.^23^ While tissue-engineered heart valves have been tested in limited clinical studies since the beginning of the 2000s,^23^ there is still a need for basic understanding on the tissue process, as well as the outcomes and mechanical function, before this technology can undergo regulatory approval.^24^ These two developing technologies are presented in the right panel of Figure 1.

## TRANSCATHETER AORTIC VALVE IMPLANTATION

Transcatheter aortic valve implantation is an alternative to surgical aortic valve replacement (SAVR). A stented bioprosthetic valve is delivered via catheterization and deployed inside the stenotic native valve.^25^,^26^ In the latest generation of devices, the only US Food and Drug Administration (FDA)-approved TAVI devices are the balloon-expandable Sapien 3 Ultra (Edwards Lifesciences Corp., Irvine, CA, USA), the self-expandable Evolut Pro (Medtronic, Minneapolis, MN, USA), and the self-expandable and mechanically locked Lotus Edge (Boston Scientific, Marlborough, MA, USA).^27^ In addition to these three devices, numerous other TAVI devices (including the Portico from Abbott, the Acurate Neo from Boston Scientific, and JenaValve’s device) have received Conformité Européenne (CE) marks^16^; however, most of them have been discontinued. While each of the various CE-marked devices has its own advantages, these advantages are usually related to aspects other than valve hemodynamics. Although TAVI was originally approved for only high-risk surgical patients, it is now approved for low-risk surgical patients.^28^ This recent change could increase the annual number of TAVI procedures from 180,000 to 270,000 in Europe and North America.^29^ In addition to the classical use of TAVI in CAVD, both self-expanding and balloon-expandable TAVI devices are FDA-approved for ViV implantation.^30^ However, the suture ring of the SAVR makes it narrower than the native root, and inserting a TAVI reduces the orifice area of the valve even more, thereby harming hemodynamic performance.^31^ Additionally, some of the TAVI complications are more common in ViV (discussed in the next section). Transcatheter aortic valve implantation is also being used in bileaflet aortic valve patients at high surgical risk, although such use has not yet been approved for this population.^32^ The risk of various hemodynamic complications is also higher than in CAVD patients, due to the non-circular anatomical shape of the aortic valve in these patients.

### Hemodynamic Complications

The new-generation TAVI devices demonstrates a vast decrease in complications; however, the existence of some adverse outcomes remains a concern due to the shift toward use in lower-risk patients.^31^ Some of these complications include conduction abnormalities (necessitating permanent pacemaker implantation), coronary artery obstruction (CAO), paravalvular leak (PVL), and valve thrombosis.^31^ The two last-mentioned are direct hemodynamic complications.

#### Paravalvular leak

**Figure 2:**
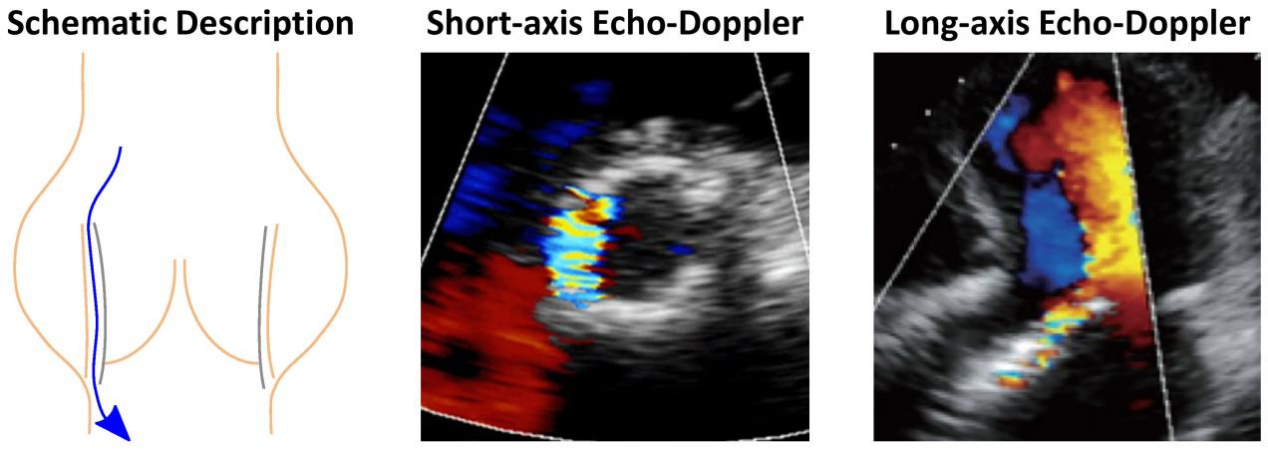
Paravalvular Leak Post-Transcatheter Aortic Valve Implantation (TAVI) Schematic description of paravalvular leak with examples of how it is seen in echo-Doppler images (adapted from Figure 3 of Di Martino et al.^33^ [CC BY 4.0] and Figure 14 of Sordelli et al.^34^ [CC BY-NC-SA]).

Paravalvular leak is a leakage through the gaps between the implanted stent of TAVI devices and the native valve (Figure 2). This adverse hemodynamic outcome has been significantly minimized in the latest-generation devices, from a prevalence in patients of 25% to 5%.^16^ This reduction was achieved by adding an outer skirt or cuff that covers the ventricular portion of the stent. In the original Sapien 3 valve, the outer skirt included openings that created pockets that could fill with blood, thereby sealing the paravalvular gaps.^16^ This design was later refined in the Sapien 3 Ultra valve by increasing the outer skirt height, closing the pockets, and adding texture to the polyethylene terephthalate fabric. While the latest self-expandable devices also have an outer skirt, they can seal the gaps with an optimized anatomical fitting, specifically by having a larger stent diameter on the ventricular side than in the valve region. In addition to design improvements in the latest devices, if aortic regurgitation (AR) is found immediately following implantation, PVL is minimized by post-dilation with ballooning. These advances significantly reduced the incidence of AR post-TAVI; however, moderate-to-severe AR is still much more frequent compared to SAVR.^35^ Since PVL is a very patient-specific complication, from an engineering point of view, it is more useful to evaluate it with computational fluid dynamics (CFD) than with bench experiments.^36^ In CFD, the basic equations that describe the flow of a fluid are solved by computational software. Therefore, CFD enables virtual replication of procedural options that cannot be tested *in vitro* for specific patients. Several studies have used CFD^37^–^41^ and fluid-structure interaction,^42^ where the fluid dynamics equations are coupled with solid mechanics models, to estimate PVL. This is also one of the features of a commercial service for pre-procedural planning based on patient-specific scans, as described below (Patient-specific Pre-procedural Planning Based on Numerical Models).

#### Thrombogenicity

**Figure 3:**
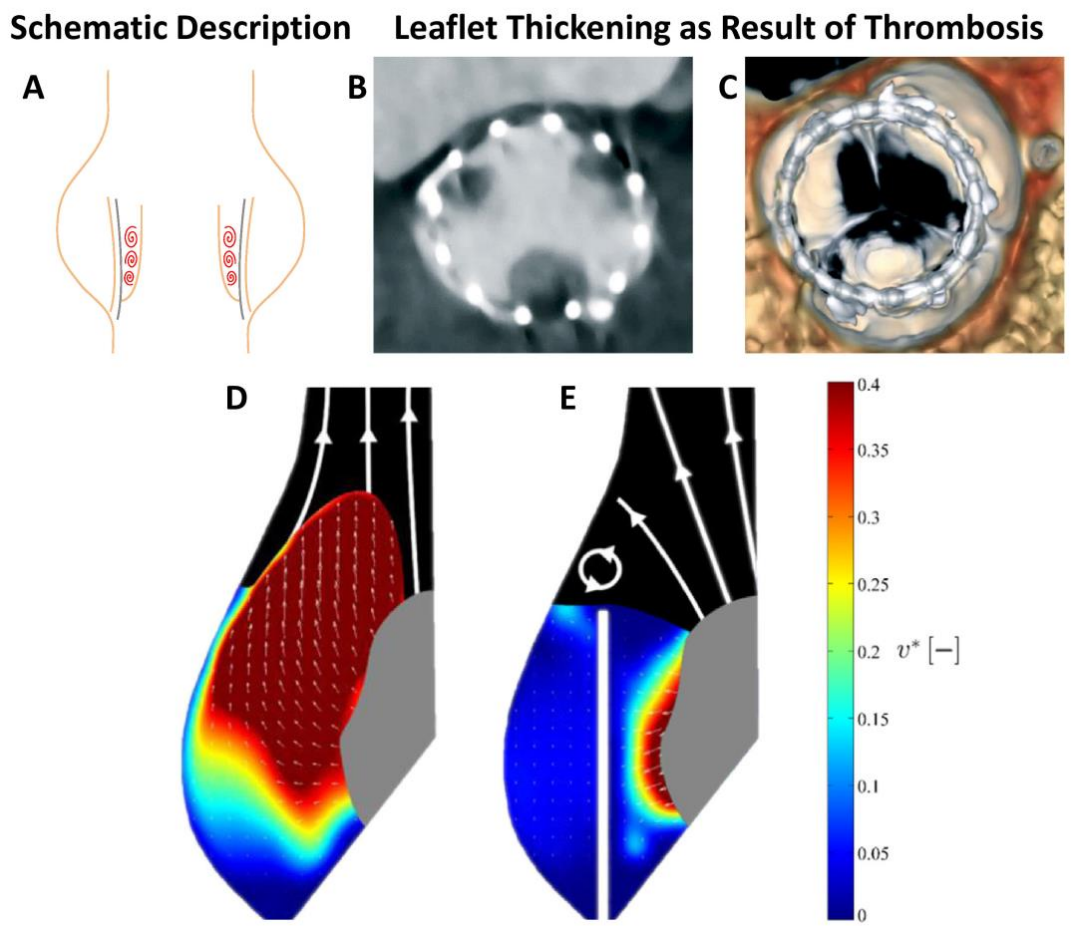
Flow Stasis Post-Transcatheter Aortic Valve Implantation (TAVI) and Leaflet Thickening That Indicates Thrombosis Location Schematic description of potential stasis **(A)**. Leaflet thickening as seen in both CT **(B)** and volume-rendered 4D-CT scans **(C)**. Blood velocity magnitude as measured from *in vitro* experiment that represents native aortic valve **(D)** and post-TAVI **(E)**. Blue shades represent stagnation (low velocities). CT scans and experimental results are adapted from Figure 2 of Rosseel et al.^49^ [CC BY 4.0] and Figure 5 of Ducci et al.^50^ [CC BY 4.0].

Hemodynamics is one of the main contributing factors for thrombosis.^43^,^44^ Exposure of platelets to elevated flow stresses and platelet adhesion in stagnant flow regions are considered the two main mechanical causes of thrombogenicity.^45^,^46^ In heart valves, these two factors, along with non-hemodynamic factors like hemocompatibility, can cause leaflet thrombosis or thromboemboli. In mechanical valves, the main concern is thrombus formation resulting from disturbed flow and elevated shear stress in the regurgitant flow through the narrow gaps. While it is true that the narrow gaps in PVL around TAVI can also cause this type of thrombus formation,^40^,^47^ obviously the leakage itself is usually a bigger concern than the thrombosis and constitutes the rationale behind performing post-TAVI dilation. On the other hand, blood flow stagnation in the valvular region is the suggested cause of leaflet thrombosis in TAVI, both in clinical studies^48^ and based on *in vitro* particle image velocimetry (PIV) measurements of the flow velocity vector field (Figure 3).^50^,^51^ Specifically, leaflet thrombosis is the assumed reason for reduced leaflet motion,^52^ as a result of hypoattenuated leaflet thickening.^53^ The prevalence of leaflet thrombosis remains unknown since cases that have not been diagnosed clinically have been discovered in pathological valve studies,^48^ but frequencies of 16% in Sapien 3, 8% in Evolut, and 14% in Lotus TAVI devices as compared with 4% in SAVR patients have been suggested.^52^ Additionally, the occurrence of leaflet thrombosis post-ViV placement was reported to be six times the occurrence of leaflet thrombosis post-TAVI in native valve.^54^

Several attempts have been made to study the hemodynamic causes of hypoattenuated leaflet thickening by engineering methods, both experimental and numerical. Experimental studies compared the native valve to TAVI,^50^ surgical valve to ViV^51^ with idealized geometry, or surgical valve to ViV with commercial valves.^55^ Numerical studies that compared surgical valve to ViV^56^–^58^ by CFD with prescribed leaflets motion also employed idealized geometry that were experimentally measured.^57^,^58^ All these studies demonstrated that valve confinement, where the TAVI valve is surrounded by the previous leaflets of the native or the degenerated SAVR valve, can increase the blood residence time near the leaflets. Therefore, supra-annular implantation, like in Evolut (where the TAVI valve is only partially confined), is expected to have a lower thrombogenic risk than a fully confined valve, such as the intra-annularly implanted Sapien.^58^ A recently proposed method to address CAO is to lacerate the leaflets of the bio-prosthetic valve by a technique known as BASILICA (bioprosthetic or native aortic scallop intentional laceration to prevent coronary artery obstruction)^59^ (see below, Hemodynamics of structural complications). In addition to the original intention of this technique, engineering studies suggest that the laceration can allow better washout and reduce the flow stagnation in the valvular region, thus leading to lower thrombogenic risk.^60^,^61^

#### Hemodynamics of structural complications

In addition to these two direct hemodynamic complications, the other TAVI complications also affect blood flow. Coronary artery obstruction obviously has a major effect on coronary hemodynamics. It is more common in ViV cases than in classical TAVI, and is related the to the surgical valve design, with a complication rate of up to 5.3% for externally mounted surgical valves.^62^,^63^ Patients suspected of being at risk for CAO, based on pre-procedural imaging, should be protected pre-emptively by “chimney” stenting.^64^ An alternative to chimney stenting is the BASILICA technique^59^ where the laceration directly prevents obstruction. While CAO risk is currently evaluated based only on the anatomic location of the coronary ostia, numerical models can help to quantitatively assess flow dynamics.^65^,^66^ The circularity and size of the valve orifice can be highly dependent on patient anatomy, especially with self-expandable TAVI devices. Clearly, it is undesirable to have a non-circular valve, and this phenomenon has been generally been addressed by the supra-annular design of the TAVI device.^16^ Finally, valve embolization (migration) exemplifies structural complications due to hemodynamics. While it is relatively rare (occurrence as low as 0.5%)^67^ and considered a consequence of insufficient anchoring, valve embolization is a direct result of the diastolic blood pressure pushing the valve into the ventricle. Recently, we suggested that BASILICA can weaken anchorage forces, although our study did not indicate that it was weakened enough to cause migration.^68^ Stronger anchoring forces, for example by over-inflating the balloon-expandable device, can obviously help prevent migration. Nevertheless, these approaches can cause additional non-hemodynamic complications such as conduction abnormalities (necessitating permanent pacemaker implantation) or even aortic root rupture.

### Patient-specific Pre-procedural Planning Based on Numerical Models

**Figure 4:**
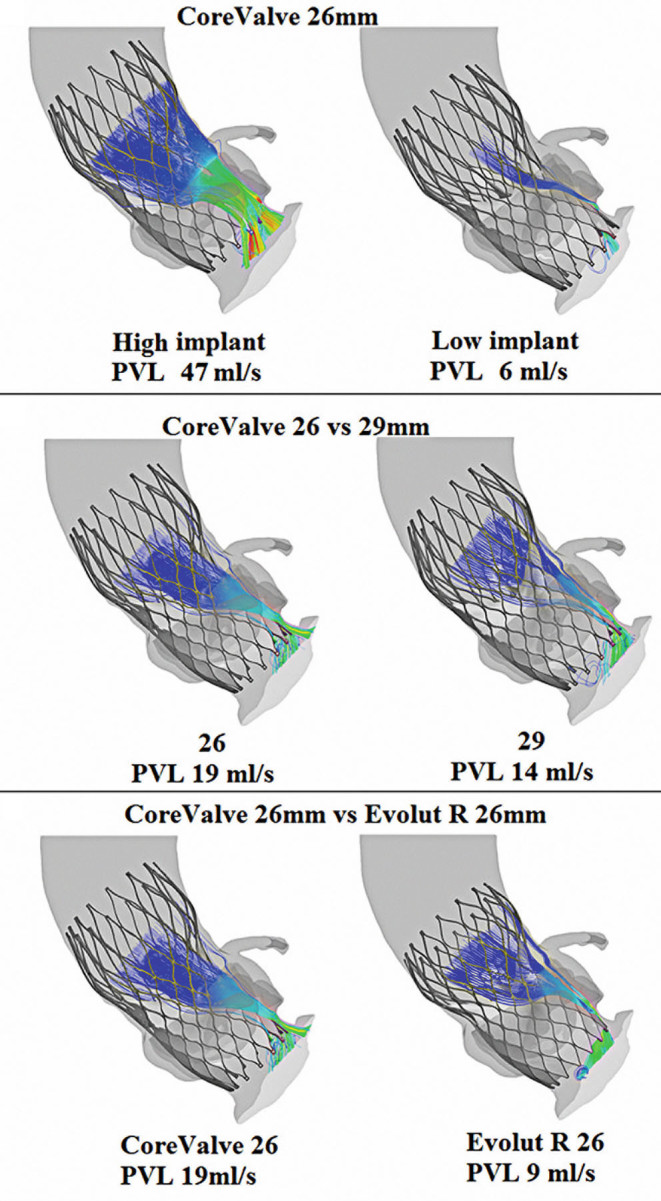
Patient-specific Pre-procedural Paravalvular Leak Calculations Examples of paravalvular leak calculations using the FEops HEARTguide. This platform can help minimize leakage by pre-selecting the optimal transcatheter aortic valve device and implantation location. Adapted from Figure 5 of El Faquir et al.^80^ [CC BY 4.0].

In recent years, several numerical models have been approved for patient-specific procedural planning in various medical treatments.^69^ The FEops HEARTguide (FEops nv, Gent, Belgium) is a CE-marked service for making pre-interventional TAVI device size and position recommendations based on patient-specific numerical models. The recommendations are based on both finite element analysis of the implantation^70^ and CFD analysis of the post-procedural PVL.^37^ To utilize this service, clinicians send routine computed tomography (CT) scans of the patient to the company. The company then reconstructs the cardiac anatomy, generates and runs the finite element analysis and CFD simulations, and provides a report on the results of several scenarios within two working days. Since the first presentation of this tool, numerous studies have demonstrated its clinical usage including for implantation in bicuspid aortic valves,^71^–^73^ and the use of TAVI in the mitral location with native calcified valves^74^,^75^ and inside a failed bioprosthetic valve.^76^ Use of the FEops HEARTguide has also been expanded to additional TAVI devices,^77^ device optimization,^78^ and also for procedural recommendations based on additional possible complications, such as conduction abnormalities.^79^ From a hemodynamic perspective, the most important capability of this tool is obviously calculation of PVL (Figure 4), which demonstrated good predictions in a clinical study of 60 patients.^37^ On the other hand, in a study that used the FEops HEARTguide to compare coronary flow with several TAVI orientations,^38^ there was no difference between the flow results in the various cases.

## THE MITRAL VALVE

Mitral valve regurgitation (MVR) is a leakage due to improper closure of the valve. It is the most common valvular heart disease with a prevalence of approximately 1.7% in the adult population.^81^ Due to its increased prevalence with age and the growing aging population, the number of cases in the year 2030 is expected to be almost double that of the year 2000.^82^ Mitral valve regurgitation is caused by either a valve prolapse (primary MVR, due to a degenerative abnormality of the leaflets, chordae tendineae, papillary muscles, or the mitral annulus) or a left ventricular dysfunction (secondary or functional MVR).^83^ Current treatments include mostly surgical valve repair or replacement.^84^ Valve replacement involves replacing the native valve with a prosthetic one. Obviously, implantation orientation significantly influences the flow pattern, especially with mechanical valves, but it is less clear what the optimal orientation is.^85^ In a valve repair, the native valve remains in place, and the leaflets, chordae, and papillary muscle are manipulated to restore normal valve behavior with a stabilized annulus while preserving the valve’s orifice size and left ventricular function.^86^,^87^ Repair techniques are based on annuloplasty, resection, addition of artificial chordae, or a combination thereof.^88^,^89^ Annuloplasty, with either rigid or flexible rings or bands, is necessary in most repairs.^90^ Valve repair is recommended for patients with primary MVR^90^ since the repair has a low mortality rate^91^ and it improves ventricular function with no need for anticoagulation. However, there remains a risk of residual MVR and concerns regarding mitral valve repair durability.^92^

Unfortunately, the majority of severe MVR patients are not treated due to the high surgical risk, leading to considerable morbidity and mortality.^93^,^94^ As a consequence, these patients can only be treated percutaneously. There is one such device that has been approved for MVR by the FDA, the MitraClip (Abbott Laboratories, Abbott Park, Illinois, USA). This transcatheter procedure involves the implantation of a clip that grasps both the anterior and the posterior leaflets of the mitral valve, mimicking surgical edge-to-edge valve repair that is done via open heart surgery.^95^ However, the procedural results are often suboptimal even in patients who meet the inclusion criteria, i.e. severely symptomatic secondary MVR patients.^96^ The Pascal system (Edwards Lifesciences Corporation, Irvine, CA), which is still in clinical trials, is another edge-to-edge valve repair device that aims to tackle some of these limitations by including wider paddles and a central spacer.^97^ Other percutaneous CE-marked interventions are based on direct (Mitralign System, from Mitralign, Inc., Tewksbury, MA, USA; Cardioband, from Edwards Lifesciences) and indirect (Carillon System, from Cardiac Dimensions, Inc., Kirkland, WA, USA) mitral annuloplasty.^95^,^98^

Transcatheter mitral valve replacement (TMVR) is a potential alternative to surgical treatment for a wide range of pathologies that cannot currently be treated percutaneously.^95^ However, TMVR has unique challenges, such as the size and shape of the valve, lack of calcification deposits for anchorage, high hemodynamic pressures, and complex sub-valvular apparatus. These challenges led to a limited clinical experience with such devices.^93^ A significant effort is being made to develop TMVR devices, with more than 10 currently at various stages of development.^99^–^101^ These TMVR devices have several mechanisms for anchorage and sufficient sealing around the device. Unlike TAVI, the main anchoring challenge focuses on not applying strong radial forces, which can obstruct and damage the aortic valve.^101^ Suggested TMVR anchoring mechanisms include counteracting axial forces by using ventricular tethers, native valve anchors, atrial and ventricular flanges, sub-annular hooks, atrial cages, and implantation of a docking system. From a hemodynamic perspective, all these anchoring mechanisms contribute to some flow disturbances. Nevertheless, flow through the valve itself should be similar to the flow through any other type of bioprosthetic valve. Another possible procedural complication with a direct hemodynamic effect is left ventricular outflow tract obstruction. For these cases, laceration of the anterior mitral leaflet to prevent outflow obstruction^102^,^103^ was suggested, a technique similar to using BASILICA in a TAVI.

**Figure 5:**
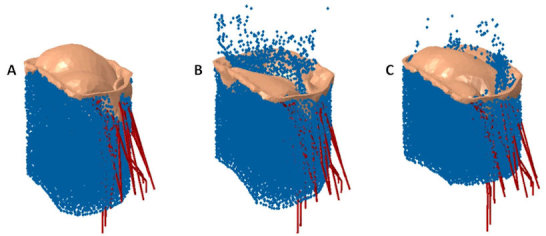
Comparison of Calculated Leakage in the Mitral Valve **A:** Healthy mitral valve. **B:** Regurgitating mitral valve. **C:** Regurgitating mitral valve post-MitraClip shows significant leakage reduction. Adapted from Figure 5 of Kamakoti et al.^122^ [CC BY 4.0].

Several studies used engineering methods to experimentally study the hemodynamics of the mitral valve, including MVR before and after treatment.^104^–^106^ Numerical methods have also been employed to model healthy and diseased mitral valves, before and after surgery.^107^–^112^ Both mitral annuloplasty^113^–^118^ and edge-to-edge procedures^119^–^121^ have been modeled with finite element analysis to evaluate their effect on tissue stress, tension in the chordae tendineae, and hemodynamics. Most numerical models of percutaneous MVR treatments focused on evaluating the commercially available MitraClip.^105^,^119^–^122^ Sturla et al.^105^ studied the effect of MitraClip implantation with both *in vitro* experiments and numerical models. While hemodynamics was not modeled numerically, it was measured experimentally. Therefore, this study was able to find a correlation between the “dry” experimental results and hemodynamics. Kamakoti et al.^122^ presented numerical simulations of fluid structure interaction in the mitral valve post-MitraClip implantation (Figure 5). Their main focus was regurgitation reduction using the MitraClip, and their results indicated the importance of the grasping location. While no study presented numerical simulations of TMVR devices, both Karady et al.^74^ and Serban et al.^75^ described use of the FEops HEARTguide to model TAVI devices (the Lotus valve from Boston Scientific, and the Sapien 3 from Edwards Lifesciences, respectively) in the mitral location. In these cases, patients suffered from severe MVR and mitral valve stenosis with significant mitral valve annulus calcification. This specific pathology enabled the use of TAVI devices rather than a dedicated TMVR device.

## THE PULMONARY AND TRICUSPID VALVES

The pulmonary and tricuspid valves are located in the outlet and inlet of the right ventricle. Obviously, the right ventricle has the same stroke volume as the left ventricle, but by exerting only one-fourth of the work and approximately one-sixth of the pressure.^123^ While the vast majority of valvular heart diseases occur in the aortic and mitral valves, there is still a large unmet need for surgical and percutaneous treatments for patients with pulmonary and tricuspid valve disorders. Treatments for these disorders need to properly function under the low hemodynamic pressure gradients of the right heart.

The two types of pulmonic disorders are stenosis, a relatively common congenital defect,^81^ and regurgitation, attributed to a variety of causes. While the gold standard treatment for pulmonic valve stenosis is balloon valvuloplasty, this treatment has long-term complications of regurgitation and restenosis.^124^ Severe regurgitation is treated by valve replacement, usually performed percutaneously. There are two FDA-approved devices for transcatheter pulmonary valve implantation: the Melody™ (Medtronic, Dublin, Ireland) and the Sapien XT (Edwards Lifesciences Corporation, Irvine, California, USA). Both devices are approved for patients with prosthetic valve regurgitation,^125^ while the Melody is also being implanted off-label in native valves.^126^,^127^ While transcatheter pulmonary valve implantation was found to have high procedural success,^125^ there are still concerns regarding complications. While most of the complications are structural, damage to the tricuspid valve was recently reported as a cause for tricuspid valve regurgitation.^128^ Only a few engineering hemodynamics studies have focused on the pulmonary valve. Suzuki et al.^129^ presented an *in vitro* experimental study of the hemodynamics in a polymeric pediatric pulmonary valve prosthesis. Capelli et al.^130^ used numerical models of patient-specific anatomies to predict the clinical outcomes of Melody valve implantation and found good agreement between their results and clinical fluid-dynamic parameters. Recently, Li et al.^131^ presented fluid–structure interaction models of pulmonary valve stenosis as a result of congenital bicuspid pulmonary valve. While the flow dynamics was modeled, most of their results focused on the geometrical differences between tricuspid and bicuspid configurations, and the only hemodynamically relevant finding was related to the geometric orifice area.

Similar to the mitral valve, tricuspid regurgitation can be treated by surgery, usually to reduce the annular dimensions, but it is recommended only in severe cases that do not have high surgical risk.^96^ Percutaneous treatment of tricuspid regurgitation is highly desirable because most patients with moderate-to-severe regurgitation cannot be treated surgically.^132^ Several percutaneous devices and techniques have been suggested for tricuspid treatments, some of which are based on adaptation of mitral valve repair. Several devices for transcatheter tricuspid annuloplasty are in development,^132^ including the Mitralign System,^133^ the 4Tech TriCinch™ (4Tech Cardio Ireland, Ltd, Galway, Ireland),^134^ and the transatrial intrapericardial tricuspid annuloplasty concept.^135^ Additionally, edge-to-edge repair of the tricuspid valve with the MitraClip device has also been suggested.^136^ Of these, engineering tools were used to evaluate only the post-procedural hemodynamics after MitraClip implantation. Vismara et al.^137^ presented an experimental *in vitro* study that tested several procedural options for transcatheter edge-to-edge repair and found it to be a viable treatment for tricuspid valve regurgitation. They compared implantation of one or two clips and considered several combinations of clipped leaflets and clipping locations. They found that grasping should involve the septal leaflet to improve both cardiac output and pressure recovery, and that the clip should be in the middle rather than a commissural position. While the two-clip procedure was effective when grasping the septal and anterior leaflets, there was no significant improvement compared with the single clip procedure. Recently, Dabiri et al.^138^ used numerical models to perform a similar study on the effect of MitraClip positioning. This study only considered the grasping of the septal and posterior leaflets with three grasping locations and the use of two clips. They concluded, similarly to Vismara et al.,^137^ that a single clip placement in the middle produced the best outcomes and that there was no further improvement by the second clip.

## HEMODYNAMIC STANDARDS FOR TESTING PROSTHETIC VALVES

Regulatory approval of a new prosthetic valve requires demonstration of adequate hemodynamic performance. The current standards for TAVI devices (International Organization for Standardization [ISO] 5840-3) and surgical valves (ISO 5840-2) describe the experimental methods and minimum requirements for hemodynamic performance. For example, the effective orifice area of TAVI valves is required to be larger than that of comparable size SAVR valves. This requirement is a direct consequence of the thinner stent relative to the surgical suture ring, which increases the opening area of TAVI. In addition to the ISO standard, there is a trend toward *in vitro* experiments with patient-specific anatomies.^20^,^139^–^148^ This trend paves the way to more physiologic results, which until now could only be numerically simulated.^16^,^144^ Therefore, patient-specific experiments can also provide a better platform for validation of numerical models. This is specifically important with the current advances in pre-procedural planning (see Patient-specific Pre-procedural Planning Based on Numerical Models, above) and the increased demand by regulatory agencies to rely on computational modeling for device approval.^149^ The main reason for this requirement is that numerical models can still capture hemodynamic details that cannot be measured by any other means,^150^ such as the 3D velocity vector field or the spatial distribution of shear stress. In an attempt to standardize the numerical modeling methodologies and their credibility, the FDA became involved with the American Society of Mechanical Engineers (ASME) Committee for the new verification and validation standard for computational modeling of medical devices (ASME V&V 40).

## SUMMARY

This paper reviewed state-of-the-art clinical and engineering advancement in heart valve treatments, with a focus on hemodynamics. Since the beginning of the twenty-first century, the field has been rapidly changing with the introduction of transcatheter heart valve implantation and repair. This review has looked at the treatment of heart valve disorders and several advancements currently under development or with limited use, which, in the near future, will potentially revolutionize their treatment. Examples of some of these devices include flexible polymeric valves, patient-specific pre-procedural planning based on numerical models, and the expansion of transcatheter valve implantation to the mitral, pulmonary, and tricuspid valves. These advancements, and more gradual enhancements in procedural techniques and imaging modalities, could improve the quality of life of patients suffering from valvular disease who cannot, currently, be treated.

## Abbreviations

AR: aortic regurgitation
ASME: American Society of Mechanical Engineers
BASILICA: bioprosthetic or native aortic scallop intentional laceration to prevent coronary artery obstruction
CAVD: calcific aortic valve disease
CAO: coronary artery obstruction
CE: Conformité Européenne
CFD: computational fluid dynamics
CT: computed tomography
FDA: Food and Drug Administration
ISO: International Organization for Standardization
MVR: mitral valve regurgitation
PVL: paravalvular leak
SAVR: surgical aortic valve replacement
TAVI: transcatheter aortic valve implantation
TMVR: transcatheter mitral valve replacement
US: United States
ViV: valve-in-valve
